# A coherent method for combined stable magnesium and radiogenic strontium isotope analyses in carbonates (with application to geological reference materials SARM 40, SARM 43, SRM 88A, SRM 1B)

**DOI:** 10.1016/j.mex.2020.100847

**Published:** 2020-03-03

**Authors:** Jessica A. Stammeier, Oliver Nebel, Dorothee Hippler, Martin Dietzel

**Affiliations:** a Institute of Applied Geosciences, Graz University of Technology, Rechbauerstraße 12, 8010 Graz, Austria; b GFZ German Research Centre for Geosciences, Telegrafenberg, 14473 Potsdam, Germany; c School of Earth, Atmosphere and Environment, Monash University, Clayton VIC 3800, Australia

**Keywords:** Mg isotopes, Radiogenic Sr, MC-ICP-MS, Certified, carbonate-bearing reference material, Chemical separation

## Abstract

We undertook ^87^Sr/^86^Sr analyses for a range of carbonate bearing geological reference materials, and combined these with δ^26^Mg for a subset of samples. Following chemical purification in a series of chromatographic extractions, isotope ratios were measured by Multi-Collector-ICP-MS using a Plasma II (Nu instruments, Wrexham, UK). To validate efficient sample digestion procedures of carbonate fractions, total samples were treated with either 3 mol l^−1^ HNO_3_ and 0.5 mol l^−1^ HCl, respectively. Results of both leaching procedures are identical within reproducibility. Reference values for SRM 88A (formerly NBS 88A), SRM 1B (formerly NBS 1B), SARM 40, SARM 43, JDo-1, JLs-1, and San Carlos olivine range from 0.70292 to 0.73724 in ^87^Sr/^86^Sr and from -2.80 to -0.41 ‰ for δ^26^Mg, respectively. This set of geological reference materials can be used for sedimentary rock material with different carbonate mineral and matrix composition as quality control measurements of combined stable Mg and radiogenic Sr isotope analyses.

• We present a protocol that facilitates the chemical separation of Mg and Sr in carbonate bearing geological reference materials including ^87^Sr/^86^Sr and δ^26^Mg of certified reference materials.

## Graphical abstract

**Figure:**
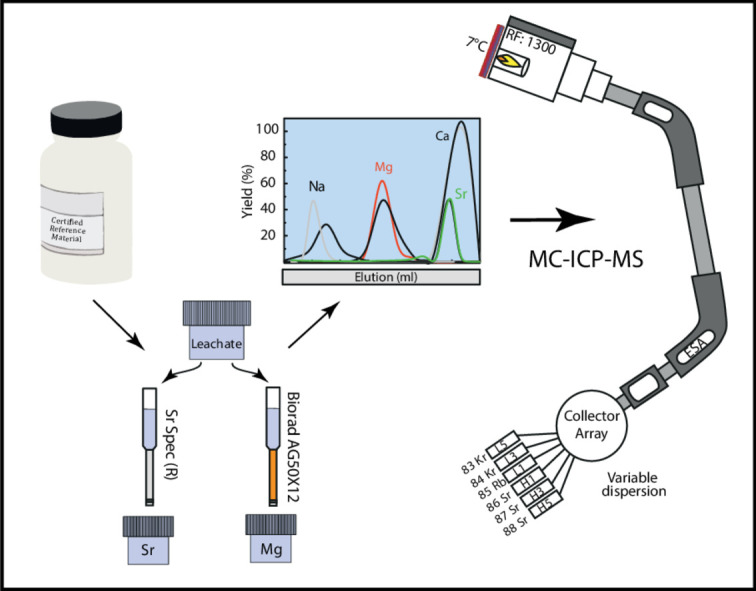

**Table:** Specification Table

Subject Area:	Earth and Planetary Sciences
More specific subject area:	*Isotope geochemistry*
Method name:	*Coupled Magnesium and Strontium separation for isotope analysis*
Name and reference of original method:	
Resource availability:	

## Method details

  

## Introduction

Studies on stable and radiogenic isotope variations in natural materials have substantially increased over the last decades and, together with technological and scientific development, provide nowadays high precision analyses for a large number of isotopic systems (e.g., [1], [2], [3]). High-precision isotope analyses have become a cornerstone of scientific research with applications in the fields of hydro- and geosciences as well as e.g., forensics, archaeology or medical sciences [4], [5], [6]. Such analyses, however, require means of testing accuracy and precision as well as newly established methodologies in laboratories.

Among the non-traditional stable isotope systems that of Mg is of particular interest, because it is an important element in most natural surroundings. For low-temperature environmental processes, Mg isotope analyses are traditionally employed to trace source of fluid-borne Mg or to study process related elemental and isotope fractionation mechanisms and kinetics. The latter include (1) fluid-rock interaction, e.g., during weathering, soil formation, mineral surface reactions and dissolution – re-precipitation reactions [7], [8], [9], [10], [11], [12] as well as (2) biologically controlled processes, such as formation and decomposition of organic substances, biomineralization or ion transport through cell membrane channels [13], [14], [15], [16], [17]. In low-temperature settings, the ^26^Mg/^24^Mg variation is in the 5 ‰ range [18], [19], [20], [21], exceeding reported reproducibility tenfold (e.g., [13,22]).

Among radiogenic isotope systems, ^87^Sr/^86^Sr is well-established in low-temperature marine research, in particular considering carbonate, phosphate and sulphate minerals. For the latter mineral groups, the incorporated radiogenic Sr in the bivalent ion position of the mineral structure is used as an environmental proxy and tracer. Accordingly, radiogenic Sr isotopes have been used to trace Sr sources and mixing behaviour in aquatic bodies [23], [24], [25]. Globally, by means of the relatively long residence times in ocean water, Sr isotopes are further considered to be almost homogeneously distributed in global oceans over a million-year time-interval, which has led to the well-established Phanerozoic seawater ^87^Sr/^86^Sr evolution curve. Recorded variation in past ocean waters from ca. 0.710 to 0.706 [26], [27], [28] can thus potentially be used to trace silicate weathering vs*.* mid-ocean ridge hydrothermal influx [29], and through Sr chronostratigraphy may provide rough age constraints when compared with the seawater ^87^Sr/^86^Sr evolution [30].

Combining stable Mg isotope and radiogenic Sr data has great potential within multi-proxy approaches, in particular in low temperature environments due to their high abundance in aquatic systems, solely divalent ion character and complementary stable vs*.* radiogenic isotope tracer behaviour. Whilst Sr isotopes can routinely be analysed through thermal ionisation mass spectrometry (TIMS), Mg isotopes can be performed to much higher efficiency with a multi-collector inductively couple plasma mass spectrometer (MC-ICP-MS). However, despite pitfalls [31], Sr has also been analysed with MC-ICP-MS providing a much higher sample throughput [32].

Chemical separation protocols for both Mg and Sr have been tested and optimised for different matrices, using cation exchange resin, e.g.,: AG50W-X12 (BioRad^Ⓡ^, Hercules, USA), for Mg separation and Sr specific chromatographic resin, e.g., from Eichrom Technologies Inc. (USA) or TrisKem International (France), for Sr separation (e.g., [21,33]). Some of these protocols facilitate the simultaneous separation of different elements [22,^33^,34]. Among these, simultaneous separation of Mg and Sr (and also Ca) is especially interesting for carbonate bearing materials. Although, the use of combined radiogenic and stable isotope investigations, e.g., in Proterozoic to Phanerozoic carbonate rock, requires reference material for quality control measurements for testing accuracy and precision, surprisingly little combined δ^26^Mg-^87^Sr/^86^Sr isotope data are available for carbonate bearing geological reference materials.

In this study, we carried out combined stable Mg – radiogenic Sr isotope analyses on natural calcareous and carbonate bearing geological reference materials using MC-ICP-MS, where separation protocols were modified after [22]. Our protocols are developed in order to facilitate the near simultaneous or coupled routine analyses of Mg-Sr isotopes in Ca-rich samples. Here, we employ two different digestion methods for carbonate rocks (e.g. limestones and dolostones), using 0.5 mol l^−1^ HCl and 3 mol l^−1^ HNO_3_, respectively. We analysed the HCl and HNO_3_ soluble fraction of geological reference materials that include the carbonate minerals calcite, dolomite and/or magnesite.

## Materials and methods

### Reference materials

For both isotope systems, respective Mg and Sr isotope values were determined on different types of calcareous and carbonate bearing geological reference materials and seawater. Reference materials SRM 88A, SRM 1B, SARM 40, SARM 43, JDO-1, and JLS-1 were chosen to represent carbonate materials with varying Ca/Mg ratios (0.02–127) and different bulk mineral chemistry (Table 1). Additionally, for Mg isotopes, the non-certified reference material “San Carlos olivine” was analysed, a natural, forsterite-rich olivine with reported isotope values for Mg [35]. For quality control, reference materials IRMM-009 and Cambridge-1 (CAM-1) were analysed. These two reference materials are pure Mg nitrate solutions that were prepared in batch and distributed by the Institute for Reference Materials and Measurements (IRMM-009) and A. Galy (CAM-1) [36,37]. A brief sample description is presented in Table 1.

**Table 1:** Sample description including selected element abundances of the analysed certified geological reference materials used in this study.

Reference material	Sample description	Distributer	Element abundances				Sampling site/References
			MgO (wt.%)	CaO (wt.%)	Sr (µg g^−1^)	Ca/Mg	
SRM 88A (*former NBS 88A)	Dolomitic limestone	NIST	21.3	30.2	85.0	1.42	Certificate of NIST SRM88A (1982)
SRM 1B (*former NBS 1B)	Argillaceous limestone	NIST	0.36	50.9	1180	127	Certificate of NIST SRM 1B (1966)
SARM 40	Carbonatite	SARM	1.97	49.8	1600	25.3	a
SARM 43	Magnesite	SARM	44.1	0.75	8.00	0.02	a
JDO-1	Dolomite	GSJ	18.5	34.0	56,116	1.84	b
JLs-1	Limestone	GSJ	0.62	55.0	296	88.7	a
San Carlos olivine	(gem-quality) Olivine	Natural sample					San Carlos, USA
IRMM-009	nitrate solution	IRMM					Certificate of IRMM-009 (2001)^c^
Cambridge-1	nitrate solution	A. Galy					d
Seawater	Seawater was filtered and acidified prior to analyses						Kiel Förde, Baltic Sea

NIST – National Institute of Standards and Technology (USA); SARM – South African Bureau of Standards (South Africa); GSJ - Geological Survey of Japan (Japan).

a K. Govindaraju, 1994 Compilation of working values and descriptions for 383 geostandards., Geostand. Newsl. 118 (1994) 1–158. https://doi.org/10.1046/j.1365-2494.1998.53202081.x-i1.

b N. Imai, S. Terashima, S. Itoh, A. Ando, 1996 Compilation of Analytical Data on Nine GSJ Geochemical Reference Samples, “Sedimentary Rock Series,” Geostand. Geoanalytical Res. 20 (1996) 165–216. https://doi.org/10.1111/j.1751-908X.1996.tb00184.x.

c European Commission, IRMM reference materials catalogue, (2015). http://irmm.jrc.ec.europa.eu/reference_materials_catalogue/catalogue/Pages/index.aspx (accessed February 10, 2020).

d A. Galy, O. Yoffe, P.E. Janney, R.W. Williams, C. Cloquet, O. Alard, L. Halicz, M. Wadhwa, I.D. Hutcheon, E. Ramon, J. Carignan, Magnesium isotope heterogeneity of the isotopic standard SRM980 and new reference materials for magnesium-isotope-ratio measurements, J. Anal. At. Spectrom. 18 (2003) 1352. https://doi.org/10.1039/b309273a.

### Purification of chemical reagents

Sample digestion and ion (-exchange) chromatographic separation of Mg^2+^ and Sr^2+^ were carried out in laminar flow hoods, using Savillex^Ⓡ^ or AHF ^Ⓡ^ PFA beakers. Both HNO_3_ and HCl acids (*pro analyses* quality) used for separation and dilution were doubly purified by sub-boiling distillation in a PFA Savillex^Ⓡ^ DST-1000. The blanks of the purified acids were tested to be below the detection limits of < 50 ng l^−1^ for both Mg and Sr. Dilution of acids was performed with 18.2 MΩ* cm H_2_O (at 25 °C with <5 ng ml^−1^ TOC; MilliQ^Ⓡ^). Beakers were cleaned in a two-step cleaning process involving both boiling in 5 mol l^−1^ HNO_3_ and 6 mol l^−1^ HCl at 120 °C for a minimum of 24 h each. Other lab equipment (e.g., PE bottles, pipette tips) were cleaned in 0.8 mol l^−1^ HNO_3_ at 60 °C for at least 48 h.

Ion chromatographic resins were alternately cleaned in MilliQ^Ⓡ^ water and 1 mol l^−1^ HNO_3_ or HCl, respectively, and then stored in MilliQ^Ⓡ^ water. For Mg separation BioRad^Ⓡ^ AG50 × 12 resin was used. Columns consist of polypropylene with 5 cm length and 0.5 mm diameter loaded with 1 ml resin and an additional 5 ml reservoir. The columns - including the resin - were cleaned in several millilitres of both acids before use. In between each cleaning step the resin was rinsed with several column volumes of MilliQ^Ⓡ^ water. Strontium columns are prepared for each separation individually. Columns and polyethylene frits were cleaned in 0.45 mol l^−1^ HNO_3_ and stored in MilliQ^Ⓡ^ water until used.

## Sample digestion

The San Carlos olivine was grinded in an agate mortar and then digested in 3 ml of a 1:3 HNO_3_–HF concentrated acid mixture, sealed tight and left to boil on a hot plate at 110 °C for 24 h. The HNO_3_–HF was evaporated at 70 °C and the samples were treated with a small amount of concentrated HNO_3_ and concentrated H_2_O_2_ as oxidizing agents to eliminate Ca-fluoride complexes. These solutions were then dried and re-dissolved in concentrated HCl to eliminate remaining nitrates. Prior to separation, seawater was filtered through a 0.45 µm membrane acetate filter (Sartorius). For chemical separation, 10 ml of seawater was dried down and treated with small amounts of H_2_O_2_ and concentrated HNO_3_ to break up potential organic complexes.

For the geological reference materials SRM 88A, SRM 1B, SARM 40, SARM 43, JDO-1, and JLS-1, ca. 100 mg powdered material was digested. From these stock solutions, aliquots were taken (different in volume), each for Mg and Sr separation, aiming for a concentration of ca. 2- 20 µg ml^−1^ for Mg depending on the Ca/Mg ratio and 20 µg ml^−1^ for Sr, and. Each sample was treated with the respective acid until no carbonate dissolution reaction was visible. Stammeier et al. [28] have shown that sample digestion using 0.1 and 3 mol l^−1^ HNO_3_ with carbonate-bearing material has no significant effect on Sr isotope composition. Thus, this method was used to evaluate (external) reproducibility and repeatability employing digestion using solely 3 mol l^−1^ HNO_3_. As incomplete digestion is especially important for Mg isotopes, we further digested a set of samples using diluted HCl for comparison. These samples were digested in 0.5 mol l^−1^ HCl, in order to evaluate internal reproducibility. Aliquots were evaporated to dryness and re-digested in the respective acids (1.5 mol l^−1^ HNO_3_ for Mg and 3 mol l^−1^ HNO_3_ for Sr) used for the respective chemical separation protocol (Tables 3, 4). One procedural blank sample was included per ten samples per separation.

## Chromatographic purification

### Magnesium separation

**Fig. 1:**
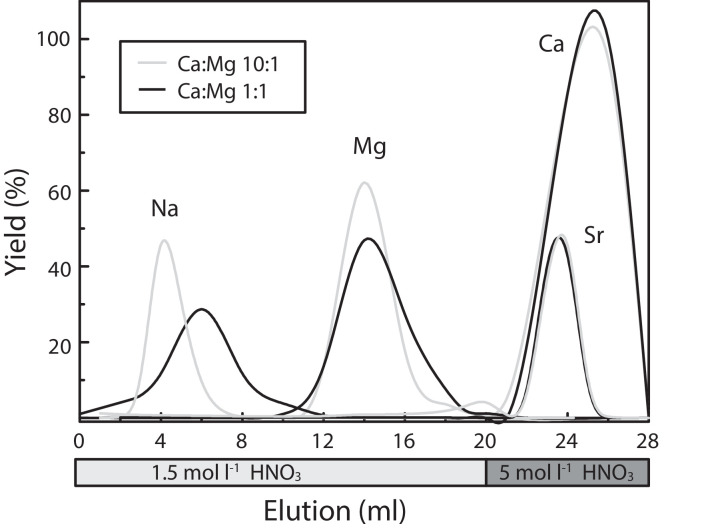
Magnesium yield during the Ca separation step. Separation was performed with two different replicates yielding identical results. After 15 ml HNO_3_ more than 99% of Mg is eluted, as is required to avoid Mg fractionation during chemical separation [33]. Calcium and Sr can effectively be eluted with a higher concentrated acid. Sodium (Na) is eluted prior to Mg, however in Na-rich samples, e.g. seawater or experimental fluids, the Na elution is retarded and overlaps with the Mg peak (not shown here).

**Fig. 2:**
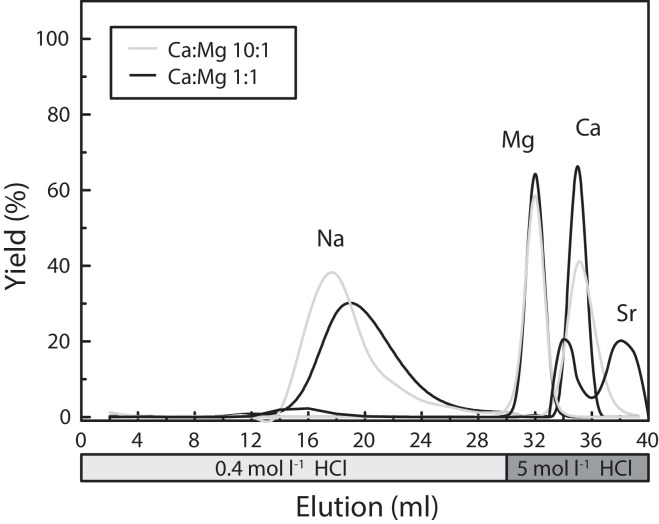
Second step of the Mg purification. The elution of Mg in an artificial solution containing Mg, Ca, Sr, and Na is identical for variable Ca/Mg (see text for details). Note that for presentation purposes each step was performed with a fresh standard solution.

For Mg purification, a two-step ion exchange chemistry was employed using HNO_3_ and HCl as eluent (after [20,22]). This two-step separation is optimized for samples with a high Ca/Mg ratio. The first step ensured the effective separation of Ca from the matrix and can also be employed for a simultaneous isolation of Zn, Fe and Ca ions for subsequent isotope analyses ([22]; Table 3, Fig. 1). In the second step, Mg was separated from other matrix elements, such as Na, K and Ti (Table 3, Fig. 2). Both separation steps were performed on the same columns using the BioRad^Ⓡ^ AG50-X12 resin. Between the two separation steps, the columns were cleaned with one column volume of 7 mol l^−1^ HNO_3_ and MilliQ^Ⓡ^ water.

Separation was tested with an artificial solution containing 10 µg ml^−1^ of Mg, Na, Sr and (i) 10 µg ml^−1^ of Ca, i.e., with a Ca:Mg ratio of 1:1; and (ii) 100 µg ml^−1^ of Ca, i.e., with a Ca:Mg ratio of 10:1. For the first Ca separation step, the columns were conditioned with 2 ml of 1.5 mol l^−1^ HNO_3_. The sample was subsequently loaded with 1 ml of 1.5 mol l^−1^ HNO_3_. After elution with 8 ml of 1.5 mol l^−1^ HNO_3_, Mg was recovered in 11 ml of 1.5 mol l^−1^ HNO_3_. The remaining divalent cations on the columns were washed off using 10 ml of 7 mol l^−1^ HNO_3_. In the second step the columns were conditioned with 0.4 mol l^−1^ HCl and loaded with the sampled or collected fraction of separation step 1. After elution of matrix elements with 30 ml of 0.4 mol l^−1^ HCl the Mg fraction was finally recovered in 4 ml of 5 mol l^−1^ HCl. Yields and potentially interfering elements were routinely tested.

### Strontium separation

For the Sr separation a single, well-established extraction ion chromatographic chemistry was employed (Table 4, after [38]). A second separation step, sometimes required for high-Rb samples [39] is not required, as calcareous or carbonate bearing material can be expected to have low to negligible Rb/Sr. The columns consisted of polypropylene pipette tips (Eppendorf) and 20–60 µm polyethylene frit material (Porex Corporation, Georgia, USA) and were filled with 100 µl Sr-Specific resin (TrisKem, France). For Sr separation 3 mol l^−1^ HNO_3_ and MilliQ water were required. Dried down samples were re-dissolved in 1 ml 3 mol l^−1^ HNO_3_. The columns were conditioned in 1 ml 3 mol l^−1^ HNO_3_ and then loaded with the sample. After washing with 4 ml 3 mol l^−1^ HNO_3_, Sr was recovered in 3 ml MilliQ.

**Fig. 3:**
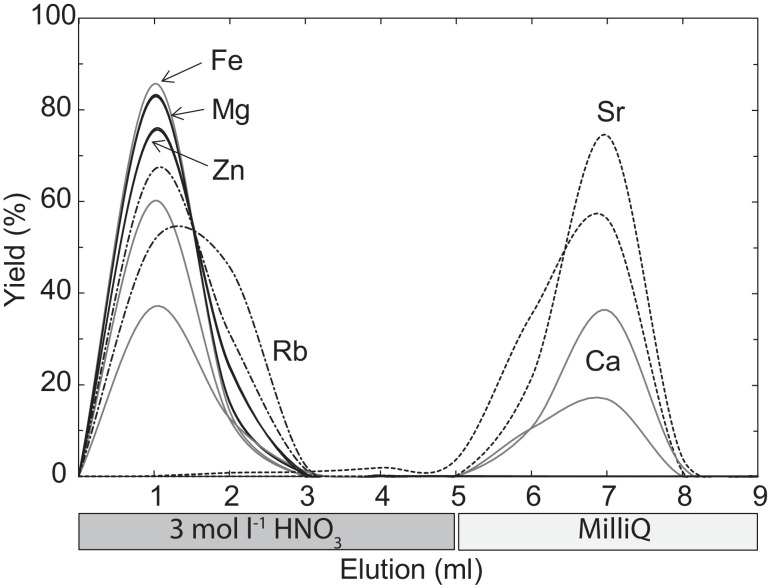
Elution curve of an artificial solution containing the elements Mg, Ca, Fe, Zn, Rb and Sr (from MERCK single element standard solution). The abscissa refers to the elution in ml with the respective solvents. Note that up to 20–40% of Ca is washed off the columns together with Sr.

To test the yield during the Sr separation, an element reference solution with 10 µg ml^−1^ of Mg, Ca, Rb, Sr, Fe and Zn (admixed from Merck single element standard solutions) was prepared (Fig. 3). The elution curve shows Rb, which forms an isobaric interference with ^87^Rb on ^87^Sr during measurements, is effectively separated after 3 ml of washing with 3 mol l^−1^ HNO_3_ and Sr should be collected after 5 ml of washing (including the ml of sample loading, Fig. 3). Further, Mg is effectively separated from the Sr fraction, potentially facilitating a coupled separation with the Sr separation step in the reverse order. However, due to the high Ca content of most samples, this is not recommended as columns may be overloaded. In fact, with Ca-rich samples some Ca is eluted together with Sr (Fig. 3), which might cause matrix effects. In these cases, the sample could be passed over the columns twice to effectively eliminate all Ca. The bulk Sr was eluted from the columns with MilliQ water.

### Data acquisition and reduction

Isotope analysis was carried out on a Plasma II MC-ICP-MS (Nu instruments, Wexham, UK) at the NAWI Central Laboratory for Water, Minerals and Rocks at Graz University of Technology, Austria. The instrumental parameters and settings during measurements of the Plasma II are summarized in Table 2 for the respective isotopes. Torch position, Ar-gas flow rates and lens set up were optimized to achieve maximum signal intensity and stability of the main beam, ^24^Mg and ^88^Sr, for Mg and Sr, respectively. Analyses were typically performed in low resolution with a sensitivity of 15 V for 150 µg l^−1^ Mg and 25 V for 500 µg l^−1^ Sr, respectively, on the highest abundant isotopes (^24^Mg, ^88^Sr). Magnesium was measured in dry-plasma mode using a DSN 100 desolvator (Nu instruments, Wrexham, UK), whereas Sr was measured in wet plasma mode using a static cup set-up. The nebulizer flow rate was 0.1 mL/min. Data acquisition of Mg and Sr isotopes consisted of 1 block with 25 cycles with an integration time of 5 s each. The background was determined by measuring 10 s at half masses before each block. To ensure repeatability and reproducibility, repeated analysis of reference materials Cambridge-1 and IRMM-009, normalised to DSM3, during Mg isotope measurements and seawater during Sr isotope measurements, were performed. Concentration of reference materials and samples was adjusted to match within 10%, in order to avoid amplification of mass bias induced differences [40]. The total procedural blank was below 0.4 µg Mg and 1.2 ng Sr and negligible compared to analyte signals. Thus, no blank correction was performed.

**Table 2:** Operation conditions for Mg and Sr isotope determination with the Plasma II MC-ICP-MS (Nu Instruments).

Parameter	Running conditions	
Analyte	Mg	Sr
RF power	1300	1300
Plasma mode	Dry mode, DSN 100	Wet mode
Auxiliary gas	0.85–0.9 min^−1^	0.85–0.95 l min^−1^
Spray chamber temperature (Peltier)	5 °C	5 °C
Nebulizer flow rate	0.1 ml min^−1^	0.1 ml min^−1^
Nebulizer type	MicroMist concentric pyrex nebulizer (GlassExpansion)	
Cone + skimmer	Ni	Ni

**Table 3:** Two-step separation protocol for Mg using Biorad AG50W-X12 resin. Both separation steps, Ca separation and Mg purification, can be performed on the same column.

Step	Amount (ml)	Molarity [mol l^−1^]	Reagent	Elution
1) Ca removal:				
condition	2	1.5	HNO_3_	
load	1	1.5	HNO_3_	
wash	8	1.5	HNO_3_	
collect	11	1.5	HNO_3_	Mg
wash	10	7	HNO_3_	Divalent cations with atomic mass >24
cleaning	3	–	H_2_O	
2) Mg purification:				
condition	2	0.4	HCl	
load	0.5	0.4	HCl	
rinse	30	0.4	HCl	
collect	4	5	HCl	Mg

**Table 4:** Strontium separation, modified after [62].

Step	Amount (ml)	Molarity [mol l^−1^]	Reagent	Elution
Condition	1	3	HNO_3_	
Load	1	3	HNO_3_	Mg, Ca, Zn, Fe, Rb
Wash	4	3	HNO_3_	
Collect	3	–	H_2_O	Sr, Ca

#### Magnesium isotopes

Magnesium isotopes were collected in Faraday cups with a set-up reported in Table S1. Instrumental mass bias was corrected for using the standard sample bracketing (SSB) method normalizing to the DSM3 reference material [41]. Mass drift of two bracketing standards (DSM3) may exceed the anticipated repeatability of ±0.25 ‰ of δ^26^Mg [22], causing samples to be artificially shifted and yield inaccurate results. To circumvent this, bracketing standards and respective enclosed samples exceeding this repeatability were discarded. Magnesium isotope ratios are reported in the δ–notation calculated relative to DSM3 reference material:

$$\delta^{x}\text{Mg}=\left(\frac{{\left({}^{X}Mg{/}^{24}Mg\right)}_{sample}}{{\left({}^{X}Mg{/}^{24}Mg\right)}_{DSM3}}-1\right)*1000,$$

with *X* referring to either ^25^Mg or ^26^Mg, respectively.

#### Strontium isotopes

During Sr isotope measurements each isotope was collected in an assigned cup as reported in Table S2. All measured Sr isotope ratios were in-run corrected for baseline, interferences (^87^Rb and ^86^Kr) and instrumental mass bias. The latter can be corrected by using the observed mass bias factor β of an invariant isotope ratio, in this case a ^86^Sr/^88^Sr = 0.1194 [42], and the exponential law. Interference correction is applied by monitoring an isotope of the respective element without isobaric interference, e.g., ^85^Rb and ^84^Kr, ^86^Kr and subtracting the mass bias corrected isotope ratios. Krypton interferences are corrected for using a value of ^86^Kr/^84^Kr = 0.3035; Rb interferences are corrected with a value of ^87^Rb/^85^Rb=0.3857 [43]. Note that through chemical purification of Sr, Rb contents should be negligible and Rb interference correction does not affect the Sr isotope analyses. The mass bias factor β is determined in an iterative calculation: interference of ^86^Kr on ^86^Sr was first subtracted, using a synthetically biased ^86^Kr/^84^Kr. For this, a β_0_ value was calculated from a non-interference corrected, measured ^86^Sr/^88^Sr value and applied to ^86^Kr/^84^Kr to simulate mass bias for this ratio. The then corrected ^86^Sr/^88^Sr from this first step was used to calculate a new β_1_ value and the process was repeated. We found that after ten iterations of consecutive mass bias- and interference-correction β converged to a constant value. The final β_10_ value was then applied to the ^87^Rb interference corrected ^87^Sr/^86^Sr. Samples were measured in blocks of 6, which were bracketed by two consecutive measurements of NBS 987. Isotope variations in radiogenic Sr isotopes were monitored and corrected for by repeated measurements of NBS 987 in each session. The correction for systematic offsets in analytical sessions was performed by normalizing the acquired data of the average of the bracketing standards measured before and after each set of samples to a reference value of ^87^Sr/^86^Sr = 0.710250 [44].

#### Elemental concentrations

Analyses of element concentrations for calibrating columns and testing yields were performed using inductively coupled plasma mass spectrometry (ICP-MS, Agilent 7500cx) at the NAWI Laboratory for Water, Minerals and Rocks, Graz University of Technology, Austria, with a measurement uncertainty generally better than ±5% on element concentrations. Samples for the elution-calibration were taken up in 0.45 mol l^−1^ HNO_3_. The instrument was tuned to achieve maximum sensitivity while maintaining low oxide production and doubly charged ion ratios with < 1.5% of the total concentrations. The concentration background was determined on a 0.45 mol l^−1^ HNO_3_ blank solution and automatically subtracted from acquired data. Instrumental drift control was performed by simultaneously running an internal reference solution with a 1 ng ml^−1^ of Sc, Ge, and Bi.

## Results and discussion

### Repeatability and reproducibility

**Fig. 4:**
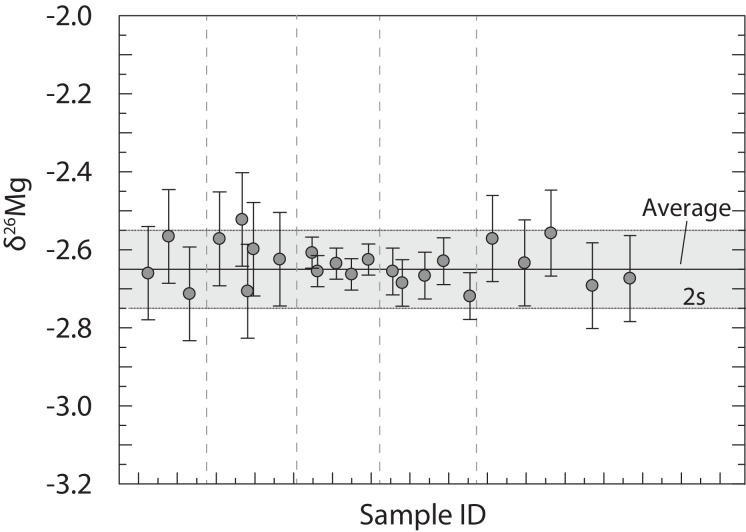
Results of CAM-1 measurements on different days, i.e., different measurement sessions, each session separated by dashed lines. In between each CAM-1 data point were usually 5–6 measurements of samples bracketed by DSM3. In this manner, external reproducibility was ensured, which was found to be ±0.10 ‰ for δ^26^Mg (2 sd). Grey area indicates the 2 sd variation of the whole data set and represents the intermediate precision of ±0.10 ‰ for δ^26^Mg. Range bars represent the repeatability measurement precision within each session and ranges from ±0.04 ‰ to ±0.12 ‰ for δ^26^Mg (2 sd).

Repeated measurements of reference solution CAM-1 yielded δ^26^Mg and δ^25^Mg, respectively, of −2.64 ± 0.10 ‰ and −1.36 ± 0.04 ‰ (2 sd, *n* = 23, *t* = 30 days; Table 5, Fig. 4). This intermediate precision (cf. IAG [45]) is identical to a reported measurement precision in the literature for these solutions of ±0.1 ‰ for δ^26^Mg (2 sd; [13,^33^,^46^,47]). Chemical separation and Mg isotope measurements of the reference material JDo-1 and IRMM 009 yielded a whole procedural reproducibility of ±0.11 ‰ and ±0.09 ‰, respectively, for δ^26^Mg (2 sd) compared to previously reported values [48], [49], [50]. This reproducibility is calculated as the standard deviation of the average of all samples compared to previously published values. Magnesium isotope values for all carbonate-bearing geological materials were identical within repeatability for both digestion methods (HNO_3_ and HCl).

**Table 5:** Magnesium isotope results from this study and published values presented relative to DSM3. n refers to the number of repeated measurements used to calculate the average δ-value and respective standard deviation (sd).

Name	δ^25^Mg (DSM3,‰)	±(2 sd)	δ^26^Mg (DSM3,‰)	±(2 sd)	N	Reference	Digestion
JDo-1	−1.30	0.10	−2.40	0.04	4	This study	3 mol l^−1^HNO_3_
	−1.30	0.02	−2.47	0.08	3	This study	3 mol l^−1^HNO_3_
	−1.22	0.11	−2.38	0.11	3	This study	0.5 mol l^−1^HCl
average	−1.29	0.11	−2.42	0.11	10	This study	HCl & HNO_3_
	−1.25 – −1.21	0.06–0.05	−2.40 – −2.36	0.06–0.08	3	a^,^^b^	
San Carlos olivine	−0.24	0.06	−0.41	0.09	10	This study	1:3 HNO_3_–HF
	−0.38 – −0.30	0.04	−0.73 – −0.62	0.06–0.1	4	c	
	−0.38–0.28	0.1–0.2	−0.64 – −0.58	0.15–0.31	16	d	
	−0.03	0.04	−0.06	0.07	5	e	
SRM 88A	−0.85	0.06	−1.59	0.09	4	This study	3 mol l^−1^HNO_3_
	−0.86	0.12	−1.53	0.07	4	This study	3 mol l^−1^HNO_3_
	−0.81	0.05	−1.62	0.04	3	This study	0.5 mol l^−1^HCl
average	−084	0.09	−1.55	0.10	11	This study	HCl & HNO_3_
SARM 43	−1.36	0.03	−2.77	0.12	3	This study	0.5 mol l^−1^HCl
	−1.38	0.05	−2.80	0.12	3	This study	0.5 mol l^−1^HCl
average	−1.37	0.05	−2.78	0.12	6	This study	0.5 mol l-1HCl
IRMM009	−2.90	0.10	−5.80	0.09	3	This study	
	−2.87	0.01	−5.74	0.02	15	f	
Seawater	−0.42	0.08	−0.79	0.07	3	This study	
	−0.43	0.06	−0.83 – −0.82	0.06–0.09	116	g^,^^h^, and references therein	
CAM 1	−1.36	0.04	−2.64	0.10	23	This study	
	−1.32	0.07	−2.61	0.06	12	b	

a V. Mavromatis, Q. Gautier, O. Bosc, J. Schott, Kinetics of Mg partition and Mg stable isotope fractionation during its incorporation in calcite, Geochim. Cosmochim. Acta. 114 (2013) 188–203. doi:10.1016/j.gca.2013.03.024.

b V. Mavromatis, P. Meister, E.H. Oelkers, Using stable Mg isotopes to distinguish dolomite formation mechanisms: A case study from the Peru Margin, Chem. Geol. 385 (2014) 84–91. doi:10.1016/j.chemgeo.2014.07.019.

c F.-Z. Teng, M. Wadhwa, R.T. Helz, Investigation of magnesium isotope fractionation during basalt differentiation: Implications for a chondritic composition of the terrestrial mantle, Earth Planet. Sci. Lett. 261 (2007) 84–92. doi:10.1016/j.epsl.2007.06.004.

d N.J. Pearson, W.L. Griffin, O. Alard, S.Y. O'Reilly, The isotopic composition of magnesium in mantle olivine: Records of depletion and metasomatism, Chem. Geol. 226 (2006) 115–133. doi:10.1016/j.chemgeo.2005.09.029.

e U. Wiechert, A.N. Halliday, Non-chondritic magnesium and the origins of the inner terrestrial planets, Earth Planet. Sci. Lett. 256 (2007) 360–371. doi:10.1016/j.epsl.2007.01.007.

f K. Ra, H. Kitagawa, Magnesium isotope analysis of different chlorophyll forms in marine phytoplankton using multi-collector ICP-MS, J. Anal. At. Spectrom. 22 (2007) 817. doi:10.1039/b701213f.

g G.L. Foster, P.A.E. Pogge von Strandmann, J.W.B. Rae, Boron and magnesium isotopic composition of seawater, Geochemistry, Geophys. Geosystems. 11 (2010) n/a-n/a. doi:10.1029/2010GC003201.

h M.-X. Ling, F. Sedaghatpour, F.-Z. Teng, P.D. Hays, J. Strauss, W. Sun, Homogeneous magnesium isotopic composition of seawater: an excellent geostandard for Mg isotope analysis, Rapid Commun. Mass Spectrom. 25 (2011) 2828–2836. doi:10.1002/rcm.5172.

For Sr intermediate precision, expressed as, was evaluated using seawater as a secondary reference with an uncertainty of ^87^Sr/^86^Sr = ±0.000011 (2 sd, *t* = 120 days, *n* = 19). Repeatability of ^87^Sr/^86^Sr in seawater within each session was typically within 100 ppm and thus well within reported performances of the Plasma II MC-ICP-MS [44]. Reproducibility of the whole procedure, e.g. determined on certified reference materials (CRM) JDo-1 and JLs-1 was within 50 ppm, with the exception of JDo-1 dissolved in 0.5 mol^−^*^l^* HCl with a reproducibility of only 150 ppm. However, comparison of all other ^87^Sr/^86^Sr derived from sample leaching in HCl or HNO_3_ shows only a small offset better than 50 ppm.

### Magnesium isotopes

**Fig. 5:**
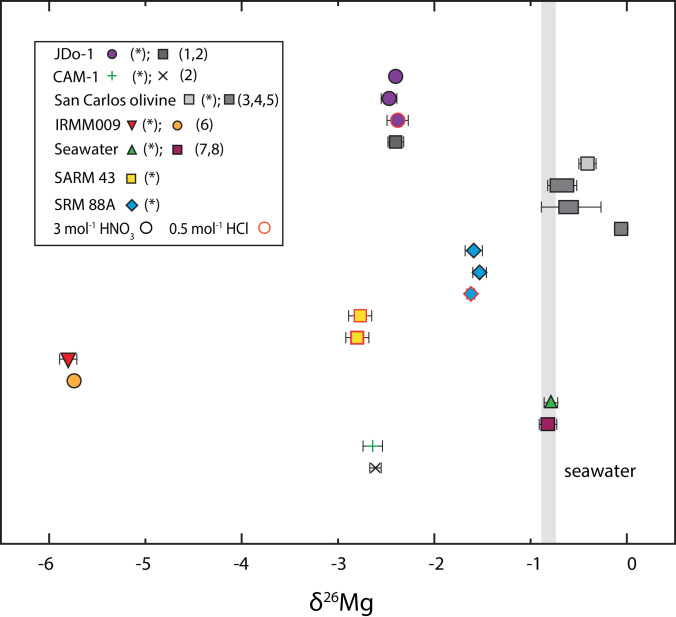
δ^26^Mg of all presented reference materials. Values labelled (*) mark results from this study. All other data points from references as follows: (1) [49]; (2) [48]; (3) [59]; (4) [47]; (5) [60]; (6) [50]; (7) [52]; (8) [53]. Symbols with a black margin represent sample dissolution in 3 mol l^−1^ HNO_3_, with a red margin represent sample dissolution in 0.5 mol l^−1^ HCl. Bright grey line indicates present day seawater. Error bars represent the 2 sd variation as reported in Table 5.

The geological reference material “San Carlos olivine” yielded a δ^26^Mg value of −0.41 ± 0.09 ‰ (2 sd, *n* = 10), which is in the range of published values (compare Table 5, Fig. 5; [51]). Seawater δ^26^Mg was −0.79 ± 0.07 ‰ (*n* = 3), identical to previously reported δ^26^Mg values of ca. 0.8 ‰ [52,53].

**Fig. 6:**
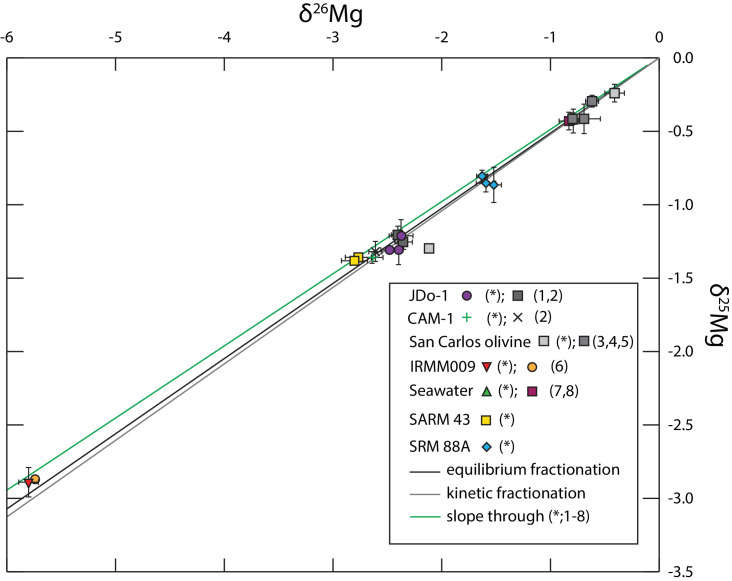
Three isotope plot displaying the δ^25^Mg vs*.* δ^26^Mg with a measured mass-dependent fractionation of β = 0.491. Note the slope of the reference data is β = 0.499 and given the resolution plots on an identical line. All data points as listed in Table 5. (*) denotes isotope data from this study. All other data points from references as follows: (1) [49]; (2) [48]; (3) [59]; (4) [47]; (5) [60]; (6) [50]; (7) [52]; (8) [53]. Symbols with a black margin represent sample dissolution in 3 mol *l* ^−^ ^1^ HNO_3_, with a red margin represent sample dissolution in 0.5 mol l^−1^ HCl. Bright grey line indicates present day seawater. Error bars represent the 2 sd variation as reported in Table 5.

Analysis of the geological reference materials SARM 43 (magnesite) and SRM 88A (dolomitic limestone) without yet published Mg isotope values, yielded results in the same range as marine limestones and dolostones [54]. The lowest average δ^26^Mg value was found for SARM 43 with −2.78 ± 0.12 ‰ (2 sd, *n* = 6). SRM 88A, a dolomitic limestone yielded δ^26^Mg values of −1.55 ± 0.10 ‰ (*n* = 11; compare Table 5). All results plot on a mass-dependent fractionation line with a slope of β = 0.491, similar to the slope of all previously published values with β = 0.499, both similar to a theoretically calculated β for equilibrium processes of 0.512 (Fig. 6; [55]). The deviation from the theoretical equilibrium fractionation slope is mainly caused by IRMM009 and SARM43, highlighted by a Δδ^25^Mg [55] of 0.07 and 0.06 for both the Mg isotope values from literature (IRMM009; [50]) and this study. The Δδ^25^Mg, calculated as δ^25^Mg’- β*δ^26^Mg and quantifies the deviation from the equilibrium fractionation line, where a value <0.04 is generally within analytical uncertainty [52]. Omitting these Mg isotope values from slope calculation the slope is β = 0.512 and thus identical to the equilibrium fractionation. Evidently those two CRM have very low δ^26^Mg, i.e., a larger difference between bracketing standard and sample, thus causing error amplification. For these CRM a different bracketing standard could be used. However, as the internationally agreed-on reference material is DSM3 this would require recalculation of these values relative to DSM3, in which case error propagation has to be considered. This would likely outweigh the observed larger Δδ^25^Mg when using DSM3 as a bracketing standard and thus not legitimate the extra effort using different bracketing materials.

### Strontium isotopes

**Fig. 7:**
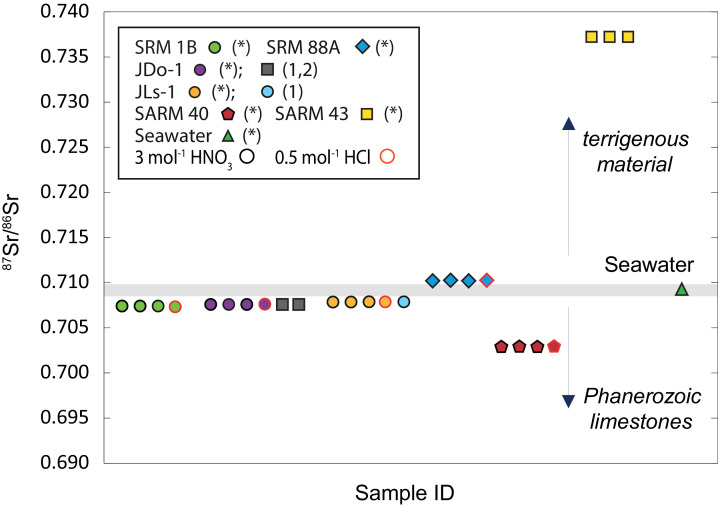
^87^Sr/^86^Sr values of certified reference materials, digested in 0.5 mol l^−1^ HCl and 3 mol^−1^ HNO_3_. The analytical uncertainty (2 sd) is smaller than symbol size. Bright grey line indicates present day seawater ^87^Sr/^86^Sr value [61].

The lowest ^87^Sr/^86^Sr of this study was analysed for the carbonatite material SARM 40 with an average value of 0.70294 ± 0.00004 (*n* = 20, Table 6, Fig. 7). Geological reference materials SRM 1B (argillaceous limestone), JDo-1 (dolomite), and JLs-1 (limestone), all have similar ^87^Sr/^86^Sr values of 0.70741 ± 0.00002 (*n* = 13), 0.70760 ± 0.00016 (*n* = 22) and 0.70784 ± 0.00002 (*n* = 14), respectively. ^87^Sr/^86^Sr values of JLs-1 and JDo-1 from this study were identical within analytical precision to published values by Miura et al. [56,57]. The dolomitic limestone SRM 88A has high ^87^Sr/^86^Sr of 0.71023±0.00004 (*n* = 12) close to the reference material used for internal normalization NBS 987. The highest value was measured in SARM 43, a magnesite, with an average value of 0.73725 ± 0.00005 (*n* = 11). The average ^87^Sr/^86^Sr value of seawater was used as an external control reference and was found to be 0.70920 ± 0.00001 (*n* = 19), identical to reported seawater values of 0.70924 ± 0.00003 (e.g., [58]).

**Table 6:** Compiled results from Sr isotope measurements; n refers to the number of analysis. All data from this study are reported relative to NBS 987 (^87^Sr/^86^Sr = 0.710250 ±0.000008 reported by [63]).

Name	^87^Sr/^86^Sr	±(2 sd)	(n)	Reference	Digestion
JDo-1	0.70756	0.000014	7	This study	3 mol l ^1^HNO_3_
	0.70756	0.000012	3	This study	3 mol l^−1^HNO_3_
	0.70757	0.000042	6	This study	3 mol l^−1^HNO_3_
	0.70767	0.000019	6	This study	0.5 mol l^−1^HCl
average	0.70760	0.000156	22	This study	HCl & HNO_3_
	0.70752	0.00002		a	
	0.707513	0.000014		b	
JLs-1	0.70784	0.000014	3	This study	3 mol l^−1^HNO_3_
	0.70784	0.000039	2	This study	3 mol l^−1^HNO_3_
	0.70786	0.000041	4	This study	3 mol l^−1^HNO_3_
	0.70784	0.000024	5	This study	0.5 mol l^−1^HCl
average	0.70784	0.000025		This study	HCl & HNO_3_
	0.70785	0.00006		a	
SRM 88A	0.71022	0.000041	4	This study	3 mol l^−1^HNO_3_
	0.71023	0.000044	3	This study	3 mol l^−1^HNO_3_
	0.71023	0.000062	3	This study	3 mol l^−1^HNO_3_
	0.71022	0.000045	2	This study	0.5 mol l^−1^HCl
average	0.71023	0.000041	12	This study	HCl & HNO_3_
SRM 1B	0.70740	0.000022	2	This study	3 mol l^−1^HNO_3_
	0.70740	0.000011	3	This study	3 mol l^−1^HNO_3_
	0.70740	0.000023	6	This study	3 mol l^−1^HNO_3_
	0.70744	0.000025	2	This study	0.5 mol l^−1^HCl
average	0.70741	0.000019	13	This study	HCl & HNO_3_
SARM 40	0.70292	0.000014	3	This study	3 mol l^−1^HNO_3_
	0.70292	0.000019	4	This study	3 mol l^−1^HNO_3_
	0.70295	0.000053	8	This study	3 mol l^−1^HNO_3_
	0.70294	0.000047	5	This study	0.5 mol l^−1^HCl
average	0.70294	0.000044	20	This study	HCl & HNO_3_
SARM 43	0.73724	0.000013	2	This study	3 mol l^−1^HNO_3_
	0.73724	0.000050	4	This study	3 mol l^−1^HNO_3_
	0.73724	0.000025	5	This study	3 mol l^−1^HNO_3_
average	0.73725	0.000054	11	This study	3 mol l^−1^HNO_3_
Modern Seawater	0.709197	0.000011	19	This study	

a T. Ohno, T. Hirata, Simultaneous determination of mass-dependent isotopic fractionation and radiogenic isotope variation of strontium in geochemical samples by multiple collector-ICP-mass spectrometry., Anal. Sci. 23 (2007) 1275–80. http://www.ncbi.nlm.nih.gov/pubmed/17998744 (accessed September 26, 2017).

b N. Miura, Y. Asahara, I. Kawabe, Rare earth element and Sr isotopic study of the Middle Permian limestone-dolostone sequence in Kuzuu area, central Japan: Seawater tetrad sffect and Sr isotopic signatures of seamount-type carbonate rocks, (2004). http://agris.fao.org/agris-search/search.do?recordID=AV20120117565 (accessed September 26, 2017).

## Summary

In the present study a reliable and fast method was developed to acquire stable Mg and radiogenic Sr isotopes of carbonate bearing geological materials with a relatively high Ca/Mg. A set of combined stable Mg and radiogenic Sr isotope values for CRM SARM 43 and SRM 88A, and additionally radiogenic Sr isotope values for SARM 40 and SRM 1B are suggested, which are readily available and can be used as secondary reference material as quality control measurements. To date no such values are available and further systematic work is suggested to build up a reliable database of geological reference material values. Effective chemical separation of both elements from the same digestion was achieved using ion specific resins BioRad 50W-X12 for Mg separation and Sr specific chromatographic resin from TrisKem for Sr separation. Intermediate precision was ±0.10‰ for δ^26^Mg and ±0.00001 for ^87^Sr/^86^Sr, derived from repeated measurements of CAM-1 and seawater, respectively. Whole procedural reproducibility was ±0.11 ‰ and ±0.09 ‰ for δ^26^Mg, determined on reference materials JDo-1 and IRMM 009. For Sr isotopes, the whole procedural reproducibility was determined on certified reference materials JDo-1 and JLs-1 and was within 50 ppm.

Respective average isotope values for all measured δ^25^Mg and δ^26^Mg, irrespective of dissolution type, were −1.37 ± 0.05 ‰ and −2.78 ± 0.12 ‰ (2 sd, *n* = 6) for SARM 43; −0.84 ± 0.09 ‰ and −1.55 ± 0.10 ‰ (2 sd, *n* = 11) for SRM 88A; and −1.29 ± 0.11 ‰ and −2.42 ± 0.11‰ (2 sd, *n* = 10) for JDo-1. For ^87^Sr/^86^Sr, values are 0.73725 ± 0.00011 (2 sd, *n* = 11) for SARM 43; 0.70294 ± 0.00009 (2 sd, *n* = 20) for SARM 40; 0.70741 ± 0.00004 (2 sd, *n* = 13) for SRM 1B; 0.71023 ± 0.00004 (2 sd, *n* = 12) for SRM 88A; 0.70784 ± 0.00002 (2s2 sd, *n* = 14) for JLs-1; and 0.70760 ± 0.00016 (2 sd, *n* = 22) for JDo-1. Except for one sample (JDo-1) the different digestion methods using HCl and HNO_3_ do not affect the isotope ratios of either Sr or Mg.

## Appendix Supplementary materials

Image, application 1

## References

[1] Woodhead J.D. (2005). Isotope ratio determination in the earth and environmental sciences: developments and applications in 2003. Geostand. Geoanalytical Res..

[2] Hoefs J. (2015). Stable Isotope Geochemistry.

[3] Teng F.-Z., Dauphas N., Watkins J.M. (2017). Non-Traditional stable isotopes: retrospective and prospective. Rev. Mineral. Geochem..

[4] Benson S., Lennard C., Maynard P., Roux C. (2006). Forensic applications of isotope ratio mass spectrometry - A review. Forensic Sci. Int..

[5] Slovak N.M., Paytan A. (2012). Applications of Sr isotopes in archaeology. Handbook of Environmental Isotope Geochemistry.

[6] Buncel E., Jones J.R. (1991). Isotopes in the Physical and Biomedical Sciences: Isotopic Applications in NMR Studies. https://www.osti.gov/scitech/biblio/5519442.

[7] Tipper E.T., Galy A., Gaillardet J., Bickle M.J., Elderfield H., Carder E.A. (2006). The magnesium isotope budget of the modern ocean: constraints from riverine magnesium isotope ratios. Earth Planet. Sci. Lett..

[8] Pokrovsky B.G., Mavromatis V., Pokrovsky O.S. (2011). Co-variation of Mg and C isotopes in late Precambrian carbonates of the Siberian platform: a new tool for tracing the change in weathering regime?. Chem. Geol..

[9] Mavromatis V., Pearce C.R., Shirokova L.S., a. Bundeleva I., Pokrovsky O.S., Benezeth P., Oelkers E.H. (2012). Magnesium isotope fractionation during hydrous magnesium carbonate precipitation with and without cyanobacteria. Geochim. Cosmochim. Acta.

[10] Pearce C.R., Saldi G.D., Schott J., Oelkers E.H. (2012). Isotopic fractionation during congruent dissolution, precipitation and at equilibrium: evidence from Mg isotopes. Geochim. Cosmochim. Acta.

[11] Schmitt A.-D., Vigier N., Lemarchand D., Millot R., Stille P., Chabaux F. (2012). Processes controlling the stable isotope compositions of Li, B, Mg and Ca in plants, soils and waters: a review. C. R. Geosci..

[12] Beinlich A., Mavromatis V., Austrheim H., Oelkers E.H. (2014). Inter-mineral Mg isotope fractionation during hydrothermal ultramafic rock alteration – Implications for the global Mg-cycle. Earth Planet. Sci. Lett..

[13] Wombacher F., Eisenhauer A., Böhm F., Gussone N., Regenberg M., Dullo W.-C., Rüggeberg A. (2011). Magnesium stable isotope fractionation in marine biogenic calcite and aragonite. Geochim. Cosmochim. Acta.

[14] Black J.R., Yin Q., Casey W.H. (2006). An experimental study of magnesium-isotope fractionation in chlorophyll-a photosynthesis. Geochim. Cosmochim. Acta..

[15] Bentov S., Erez J. (2006). Impact of biomineralization processes on the Mg content of foraminiferal shells: a biological perspective. Geochem. Geophys. Geosyst..

[16] Chang V.T.C., Williams R.J.P., Makishima A., Belshawl N.S., O'Nions R.K. (2004). Mg and Ca isotope fractionation during CaCo_3_ biomineralisation. Biochem. Biophys. Res. Commun..

[17] Saenger C., Wang Z. (2014). Magnesium isotope fractionation in biogenic and abiogenic carbonates: implications for paleoenvironmental proxies. Quat. Sci. Rev..

[18] Tipper E.T., Galy A., Bickle M.J. (2008). Calcium and magnesium isotope systematics in rivers draining the Himalaya-Tibetan-Plateau region: lithological or fractionation control?. Geochim. Cosmochim. Acta.

[19] Brenot A., Cloquet C., Vigier N., Carignan J., France-Lanord C. (2008). Magnesium isotope systematics of the lithologically varied Moselle river Basin, France. Geochim. Cosmochim. Acta.

[20] Pogge von Strandmann P.A.E.E., James R.H., van Calsteren P., Gíslason S.R.S.R., Burton K.W., Lithium (2008). magnesium and uranium isotope behaviour in the estuarine environment of basaltic islands. Earth Planet. Sci. Lett..

[21] Galy A., Bar-Matthews M., Halicz L., O'Nions R.K. (2002). Mg isotopic composition of carbonate: insight from speleothem formation. Earth Planet. Sci. Lett..

[22] Wombacher F., Eisenhauer A., Heuser A., Weyer S. (2009). Separation of Mg, Ca and Fe from geological reference materials for stable isotope ratio analyses by MC-ICP-MS and double-spike TIMS. J. Anal. At. Spectrom..

[23] Capo R.C., Stewart B.W., Chadwick O.A. (1998). Strontium isotopes as tracers of ecosystem processes: theory and methods. Geoderma..

[24] Evans J.A., Montgomery J., Wildman G., Boulton N. (2010). Spatial variations in biosphere 87 Sr/ 86 Sr in Britain. J. Geol. Soc. Lond..

[25] Bataille C.P., Bowen G.J. (2012). Mapping 87Sr/ 86Sr variations in bedrock and water for large scale provenance studies. Chem. Geol..

[26] Veizer J., Ala D., Azmy K., Bruckschen P., Buhl D., Bruhn F., Carden G.A.F., Diener A., Ebneth S., Godderis Y., Jasper T., Korte C., Pawellek F., Podlaha O.G., Strauss H. (1999). 87Sr/86Sr, δ13C and δ18O evolution of Phanerozoic seawater. Chem. Geol..

[27] Halverson G.P., Dudás F.Ö., Maloof A.C., Bowring S.A. (2007). Evolution of the 87Sr/86Sr composition of Neoproterozoic seawater. Palaeogeogr. Palaeoclimatol. Palaeoecol..

[28] Stammeier J.A., Hippler D., Nebel O., Leis A., Grengg C., Mittermayr F., Kasemann S.A., Dietzel M. (2019). Radiogenic Sr and stable C and O isotopes across precambrian-cambrian transition in marine carbonatic phosphorites of Malyi Karatau (Kazakhstan)-Implications for paleo-environmental change, geochemistry. Geophys. Geosyst..

[29] Palmer M.R., Edmond J.M. (1989). The strontium isotope budget of the modern ocean. Earth Planet. Sci. Lett..

[30] Shields G., Stille P. (2001). Diagenetic constraints on the use of cerium anomalies as palaeoseawater redox proxies: an isotopic and REE study of Cambrian phosphorites. Chem. Geol..

[31] Vroon P.Z., van der Wagt B., Koornneef J.M., Davies G.R. (2008). Problems in obtaining precise and accurate Sr isotope analysis from geological materials using laser ablation MC-ICPMS. Anal. Bioanal. Chem..

[32] Woodhead J.D. (2002). A simple method for obtaining highly accurate Pb isotope data by MC-ICP-MS. J. Anal. At. Spectrom..

[33] Chang V.T.C., Makishima A., Belshaw N.S., O'Nions R.K. (2003). Purification of Mg from low-Mg biogenic carbonates for isotope ratio determination using multiple collector ICP-MS. J. Anal. At. Spectrom..

[34] Bohlin M.S., Misra S., Lloyd N., Elderfield H., Bickle M.J. (2018). High-precision determination of lithium and magnesium isotopes utilising single column separation and multi-collector inductively coupled plasma mass spectrometry. Rapid Commun. Mass Spectrom..

[35] Fournelle J. (2011). An investigation of “San Carlos olivine”: comparing USNM-distributed material with commercially available material. Microsc. Microanal..

[36] European Commission, IRMM reference materials catalogue, (2015). http://irmm.jrc.ec.europa.eu/reference_materials_catalogue/catalogue/Pages/index.aspx(accessed February 10, 2020).

[37] Galy A., Yoffe O., Janney P.E., Williams R.W., Cloquet C., Alard O., Halicz L., Wadhwa M., Hutcheon I.D., Ramon E., Carignan J. (2003). Magnesium isotope heterogeneity of the isotopic standard SRM980 and new reference materials for magnesium-isotope-ratio measurements. J. Anal. At. Spectrom..

[38] Pin C., Bassin C. (1992). Evaluation of a strontium-specific extraction chromatographic method for isotopic analysis in geological materials. Anal. Chim. Acta.

[39] Nebel O., Scherer E.E., Mezger K. (2011). Evaluation of the 87Rb decay constant by age comparison against the U-Pb system. Earth Planet. Sci. Lett..

[40] An Y., Huang F. (2014). A review of mg isotope analytical methods by MC-ICP-MS. J. Earth Sci..

[41] Hilton R.G., Galy V., Gaillardet J., Dellinger M., Bryant C., O'Regan M., Gröcke D.R., Coxall H., Bouchez J., Calmels D. (2015). Erosion of organic carbon in the arctic as a geological carbon dioxide sink. Nature.

[42] Steiger R.H., Jäger E. (1977). Subcommission on geochronology: convention on the use of decay constants in geo- and cosmochronology. Earth Planet. Sci. Lett..

[43] de Laeter J.R., Böhlke J.K., De Bièvre P., Hidaka H., Peiser H.S., Rosman K.J.R., Taylor P.D.P. (2003). Atomic weights of the elements. Review 2000 (IUPAC technical report). Pure Appl. Chem..

[44] Hans U. (2013). High-precision strontium isotope measurements on meteorites: implications for the origin and timing of volatile depletion in the inner solar system. ETH Zürich.

[45] International Vocabulary of Metrology (2012). Basic and general concepts and associated terms. JCGM.

[46] Galy A., Belshaw N.S., Halicz L., O'Nions R.K. (2001). High-precision measurement of magnesium isotopes by multiple-collector inductively coupled plasma mass spectrometry. Int. J. Mass Spectrom..

[47] Pearson N.J., Griffin W.L., Alard O., O'Reilly S.Y. (2006). The isotopic composition of magnesium in mantle olivine: records of depletion and metasomatism. Chem. Geol..

[48] Mavromatis V., Meister P., Oelkers E.H. (2014). Using stable Mg isotopes to distinguish dolomite formation mechanisms: a case study from the Peru margin. Chem. Geol..

[49] Mavromatis V., Gautier Q., Bosc O., Schott J. (2013). Kinetics of Mg partition and Mg stable isotope fractionation during its incorporation in calcite. Geochim. Cosmochim. Acta.

[50] Ra K., Kitagawa H. (2007). Magnesium isotope analysis of different chlorophyll forms in marine phytoplankton using multi-collector ICP-MS. J. Anal. At. Spectrom..

[51] Hu Y., Harrington M.D., Sun Y., Yang Z., Konter J., Teng F.-Z. (2016). Magnesium isotopic homogeneity of San Carlos olivine: a potential standard for Mg isotopic analysis by multi-collector inductively coupled plasma mass spectrometry. Rapid Commun. Mass Spectrom..

[52] Foster G.L., Pogge von Strandmann P.A.E., Rae J.W.B. (2010). Boron and magnesium isotopic composition of seawater. Geochem. Geophys. Geosyst..

[53] Ling M.-X., Sedaghatpour F., Teng F.-Z., Hays P.D., Strauss J., Sun W. (2011). Homogeneous magnesium isotopic composition of seawater: an excellent geostandard for Mg isotope analysis. Rapid Commun. Mass Spectrom..

[54] Fantle M.S., Higgins J. (2014). The effects of diagenesis and dolomitization on Ca and Mg isotopes in marine platform carbonates: implications for the geochemical cycles of Ca and Mg. Geochim. Cosmochim. Acta.

[55] Young E.D., Galy A. (2004). The isotope geochemistry and cosmochemistry of magnesium. Rev. Mineral. Geochem..

[56] Miura N., Asahara Y., Kawabe I. (2004). Rare earth element and Sr isotopic study of the middle Permian limestone-dolostone sequence in Kuzuu area, central Japan: seawater tetrad sffect and Sr isotopic signatures of seamount-type carbonate rocks.

[57] Ohno T., Hirata T. (2007). Simultaneous determination of mass-dependent isotopic fractionation and radiogenic isotope variation of strontium in geochemical samples by multiple collector-ICP-mass spectrometry. Anal. Sci..

[58] Elderfield H. (1986). Isotope systematics the isotope systematics of Sr are summarized in Table I. 87Sr is a radiogenic, standards the Sr isotopic compositions of the commonly-used standards are listed in. Palaeogeogr. Palaeoclimatol. Palaeoecol..

[59] Teng F.-Z., Wadhwa M., Helz R.T. (2007). Investigation of magnesium isotope fractionation during basalt differentiation: implications for a chondritic composition of the terrestrial mantle. Earth Planet. Sci. Lett..

[60] Wiechert U., Halliday A.N. (2007). Non-chondritic magnesium and the origins of the inner terrestrial planets. Earth Planet. Sci. Lett..

[61] Elderfield H. (1986). Strontium isotope stratigraphy. Palaeogeogr. Palaeoclimatol. Palaeoecol..

[62] Pin C., Gannoun A., Dupont A. (2014). Rapid, simultaneous separation of Sr, Pb, and Nd by extraction chromatography prior to isotope ratios determination by TIMS and MC-ICP-MS. J. Anal. At. Spectrom..

[63] Hans U., Kleine T., Bourdon B. (2013). Rb–Sr chronology of volatile depletion in differentiated protoplanets: BABI, ADOR and all revisited. Earth Planet. Sci. Lett..

