# Supplementary material for: A coherent method for combined stable magnesium and radiogenic strontium isotope analyses in carbonates (with application to geological reference materials SARM 40, SARM 43, SRM 88A, SRM 1B)
Source: MethodsX. 2020 Mar 3;7:100847. doi: 10.1016/j.mex.2020.100847 (PMC7113622; doi:10.1016/j.mex.2020.100847)
Supplement: Supplementary file 1 [file mmc1.docx]

# Supplementary material

S1 Cup configuration for Mg measurements with a mass discrimination of 0.125 amu. No isobaric interferences from other isotopes are reported for these masses.

| Cup | L4 | H2 | H7 |
| --- | --- | --- | --- |
| Mass | 24 | 25 | 26 |
| Isotope | ^24^Mg | ^25^Mg | ^26^Mg |

S2 Cup configuration for static-cup Sr measurements, including isobaric interferences. Mass discrimination is 0.5 amu.

| Cup | L5 | L3 | L1 | H1 | H3 | H5 |
| --- | --- | --- | --- | --- | --- | --- |
| Mass | 83 | 84 | 85 | 86 | 87 | 88 |
| Isotope |  | Sr |  | Sr | Sr | Sr |
| Isobaric interferences | Kr | Kr |  | Kr |  |  |
|  |  |  | Rb |  | Rb |  |
